# Functional difficulties in children and youth with autism spectrum disorder: analysis of the 2019 Canadian Health Survey on Children and Youth

**DOI:** 10.24095/hpcdp.44.1.02

**Published:** 2024-01

**Authors:** Amy Farrow, Ahmed A. Al-Jaishi, Siobhan O’Donnell, Sarah Palmeter, Stelios Georgiades, Yun-Ju Chen, Patrick G. McPhee, Rojiemiahd Edjoc

**Affiliations:** 1 Public Health Agency of Canada, Ottawa, Ontario, Canada; 2 Department of Psychiatry & Behavioural Neurosciences, McMaster University, Hamilton, Ontario, Canada; 3 Offord Centre for Child Studies, McMaster University, Hamilton, Ontario, Canada; 4 McMaster Children’s Hospital, Hamilton, Ontario, Canada

**Keywords:** ASD, function, disability, adolescents, CHSCY

## Abstract

**Introduction:**

This study examined the prevalence of functional difficulties and associated factors in Canadian children/youth aged 5 to 17 years diagnosed with autism spectrum disorder (ASD).

**Methods:**

We analyzed data from the 2019 Canadian Health Survey on Children and Youth (CHSCY), a nationally representative survey of Canadian children/youth that used the Washington Group Short Set on Functioning (WG-SS) to evaluate functioning in six daily tasks. For each functional domain, binary outcomes were derived (no/some difficulty, a lot of difficulty/no ability). We used logistic regression to identify associations between demographic characteristics, educational experiences, and perceived mental and general health and the most common functional difficulties, namely those related to remembering/concentrating, communication and self-care. All estimates were weighted to be representative of the target population. The bootstrap method was used to calculate variance estimates.

**Results:**

Analysis of the records of 660 children/youth with ASD revealed that the most common functional difficulties were remembering/concentrating (22%; 95% CI: 18–27), communicating (19%; 95% CI: 15–23) and self-care (13%; 95% CI: 10–17). Lower perceived mental health was associated with increased functional difficulties with remembering/concentrating. ASD diagnosis at a lower age and lower perceived general health were associated with increased functional difficulty with communication. Parental expectations for postsecondary education were associated with decreased functional difficulty for self-care.

**Conclusion:**

One or more functional difficulties from the WG-SS was present in 39% of Canadian children/youth aged 5 to 17 years with ASD. Functional difficulties with remembering/concentrating, communication and self-care were most common.

**Highlights**

- Using the WG-SS, the most common functional difficulties in Canadian children/youth 5 to 17 years old who were diagnosed with autism spectrum disorder (ASD) were difficulties with memory or concentration (22%), communication (19%) and self-care (13%).
- Lower perceived mental health, younger age at ASD diagnosis, lower perceived general health and lower parental expectations for postsecondary education were associated with increased functional difficulties.
- Further research, including longitudinal data collection and more specialized measurement, is needed to identify the mechanisms and associated factors underlying functional difficulties in children/youth with ASD.

## Introduction

Autism spectrum disorder (ASD) is a neurodevelopmental disorder characterized by impaired communication and social interaction, and restricted and repetitive behaviours, interests and activities.^1^ The term “spectrum” in ASD reflects the wide range of symptoms and varying degrees of challenges experienced by those with the disorder.^2^,^3^ According to the 2019 Canadian Health Survey on Children and Youth (CHSCY), approximately 1 in 50 children/youth aged 1 to 17 years have been diagnosed with ASD.^4^ Children/youth with ASD exhibit heterogeneous communicative, social and behavioural capacities as well as diverse symptom presentations and functional abilities.^5^-^7^

When assessing functional ability, the concept of a “functional difficulty” refers to difficulties with basic activities, which may affect a child’s ability to participate in their day-to-day environment if this is unaccommodated.^8^ Functioning is influenced by the interaction between individual health conditions, such as ASD, and contextual factors, such as environmental factors (e.g. social and legal structures, built environment) and personal factors (e.g. gender, social background).^9^ This definition is based on the biopsychosocial model of disability, put forward in the *International Classification of Functioning, Disability and Health* (ICF); the ICF integrates the medical model, which views disability as a feature of the person or diagnosis, and the social model, which views disability as social problem created by a lack of accommodations in the environment.^9^ Functional difficulties are not rare, but their prevalence can vary widely in different populations, including people of all ages with ASD.^10^Examinations of these challenges within the ASD population are predominantly clinic-based or drawn from small, nonrepresentative samples, which limit the generalizability of the findings.

Data from the CHSCY provide a valuable resource for studying functional difficulties in children/youth aged 1 to 17 years, including those with ASD.^11^ The CHSCY uses the Washington Group on Disability Statistics Short Set on Functioning (WG-SS) to measure functional difficulty in the general population of children/youth. Although this tool is an internationally accepted method for identifying disability in children,^12^ it has not been validated specifically for children/youth with ASD. More intensive measures of functioning exist, but those developed specifically for children/youth with ASD typically require adaptive testing and are difficult to administer on a larger scale.^13^,^14^

There is a lack of nationally representative knowledge about the range of functional abilities in Canadian children/youth with ASD. Identifying these functional difficulties and their associated factors can help us understand the specific day-to-day challenges faced by this population, and, subsequently, better meet their service needs. Using cross-sectional survey data from the 2019 CHSCY, the objectives of this study were to estimate the prevalence of common functional difficulties in children/youth (5–17 years) diagnosed with ASD and explore factors associated with these difficulties.

## Methods

***Data***

We used data from the 2019 CHSCY, a national, cross-sectional survey administered by Statistics Canada that collected health information on children/youth aged 1 to 17 years living in private dwellings in Canada’s 13 jurisdictions (response rate 52.1%).^11^ The survey was implemented using electronic questionnaires and follow-up by phone interview between 11February 2019 and 2 August 2019. The survey was administered to the “person most knowledgeable,” usually a parent, and for simplicity we use the term “parent.” Children/youth aged 12 to 17 years were also surveyed for select questions.

The CHSCY sampling frame was created using the Canadian Child Benefit files, which as of 31 January 2019, included 98% of the Canadian population aged 1 to 17 years in the 10 provinces and 96% in the three territories. Because of the limitations of this sampling frame, children/youth living on First Nations reserves and other Indigenous settlements in the provinces, in foster homes and in institutions are excluded from the CHSCY data and therefore from our analysis.^11^ Age stratification and geographical sub-stratification were used to create a representative sample of the Canadian children/youth population. 

Statistics Canada selected 91796 children/youth and received 47 871 responses. Response rates were lower in the Northwest Territories, in Saskatchewan and in the 12- to 17-year age group. Most nonresponses were due to refusal or unsuccessful contact attempts.^11^ Sampling weights were calculated to account for out-of-scope units, nonresponse, extreme weight trimming and calibration-to-known population totals. For more information on the sampling and weighting procedures, refer to the CHSCY User Guide.^11^

The 2019 CHSCY dataset included 819 records of individuals aged 1 to 17 years with a self-reported ASD diagnosis. Of those 819 records, 660, representing 112966 children/youth, were 5 years of age and older; we used this sample for our analysis. The most common reasons for record exclusion from our analysis was that the respondent reported no ASD diagnosis (97.9% of records), information on ASD diagnosis was missing (0.02% of records) or the child was 4 years old or younger (19.4% of records for children with ASD).

***Outcome measures***

The WG-SS functional difficulty measurement set is an internationally accepted method for identifying disability in children.^12^ It was developed to measure disability in a culturally neutral and globally standardized way. The United Nations recommends this tool to assess progress towards equal treatment of people with disabilities. This task is part of the United Nation Convention on the Rights of Persons with Disabilities.^15^

Using this framework, we can measure the extent of disability in a way that allows comparison with data for other disabilities and from other jurisdictions. The WG-SS consists of six questions that assess a person’s ability to function in six basic activity domains: communicating, hearing, seeing, walking, remembering/concentrating and self-care (Table 1).^15^ For each of the WG-SS questions, the respondent is asked if they have no difficulty, some difficulty, a lot of difficulty or a complete inability (“cannot do at all”) to perform the task.

**Table 1:** Washington Group on Disability Statistics Short Set on Functioning (WG-SS) questions that assess a person’s ability to function in six basic activity domains

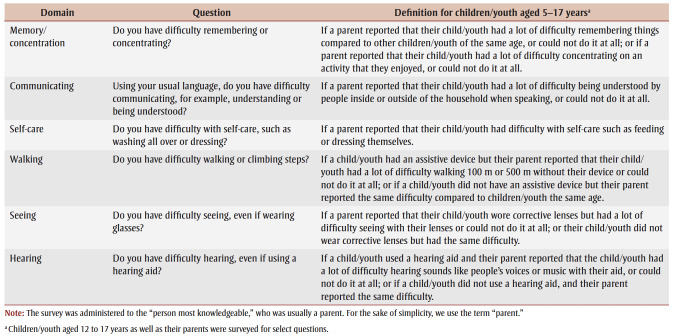

To better capture disability in children/youth, the WG-SS was adapted into a module specifically aimed at children aged 2 to 4 years and children/youth aged 5 to 17 years. This tool has been iteratively developed and validated using standard Washington Group validation procedures.^12^ The tool was not developed or tested specifically for children/youth with ASD.

Our data analyses focussed on remembering/concentrating, communicating and self-care functional difficulties because a previous analysis of the CHSCY dataset^16^ found these to be the most common among children/youth with ASD. Since remembering/concentrating and self-care functional difficulties were only defined for children/youth older than 4 years, our analysis was restricted to the population aged 5 to 17 years. Children/youth were considered to have functional difficulty when the respondent indicated that they had a lot of difficulty performing the task or were unable to perform the task. See Table 1 for the definitions for each functional difficulty.

***Factors associated with functional difficulties***

Potential associated factors were chosen from among those available in the CHSCY dataset based on a literature review targeting factors associated with daily function among children/youth and adults with ASD.^16^-^20^ We included sociodemographic variables such as sex, location of birth, racial/ethnic minority status, age, household size and household income. We also included diagnoses of neurobehavioural and mental health disorders such as attention deficit hyperactivity disorder (ADHD), anxiety disorders, mood disorders and learning disabilities. Because these disorders are characterized by inattention, impaired concentration and difficulty processing information, they are potentially associated with rates of functional difficulties.^1^

We also included academic accommodations provided to the child at school and parental expectations for the child’s future educational attainments because of the importance of academic experiences for children/youth with ASD. It is possible that children/youth with ASD with increased functional difficulties require additional academic accommodations, for example, different curricula or ways to access academic content.21 Challenges with communication combined with restricted interests and repetitive behaviours can limit the academic achievements of children/youth with ASD, and academic skills are essential for succeeding after adolescence.^22^,^23^ Youth aged 12 to 17 years reported their own academic accommodations, and parents reported for children aged 5 to 11 years. 

We included age at time of ASD diagnosis because previous studies have found that age at diagnosis differs with symptom severity.^24^-^26^ Finally, we also included two health indicators, perceived general health and perceived mental health, because of the relationship between ASD and overall health outcomes.^27^,^28^

Youth aged 12 years and older rated their own general and mental health; for children/youth where a self-rating was not available, we used the rating provided by the parent. Unless otherwise specified, all other variables used in our analyses were reported by the parent.

***Data analysis***

Following the analytical guidelines and recommendations of the WG-SS, each WG-SS functional difficulty was represented as a binary variable, where 0 represented no or some difficulty and 1 represented a lot of difficulty or no ability.^29^,^30^

We used chi-square or independent two-sample Student *t* test to compare cohort characteristics for children/youth who did and did not have remembering/concentrating, communicating and self-care functional difficulties. Multivariable logistic regression analyses were performed to understand associations between predictor variables and remembering/concentrating, communicating and self-care functional difficulties. All factors potentially associated with the outcome were included in the logistic regression.

Valid skips, where a question did not apply to the respondent and therefore was not asked, were excluded from the analyses, as were missing values. The potentially associated factors had 0% to 4% missing values, and the WG-SS variables had 0% to 1.5% (unweighted).

All estimates were weighted to be representative of the target population using sampling weights provided by Statistics Canada. Variance estimates, including 95% confidence intervals (CIs) and coefficients of variation (CVs), were determined using balanced repeated replication to account for the complex survey design. Model assumptions were met, including linearity, multicollinearity and a lack of outlier influence on the significance of the results. The observations are assumed to be independent, given the Statistics Canada sample frame definition.

An alpha criterion of 0.05 was used to determine statistical significance. Estimates with a CV of less than 15.0% were considered reliable for general use, estimates with CVs between 15.0% and 35.0% were accompanied with a warning of high sampling variability, and estimates with CVs higher than 35.0% were deemed unreliable.We conducted data cleaning steps in statistical package R version 4.1.1 (R Foundation for Statistical Computing, Vienna, AT) and statistical analyses using SAS version 9.4 (SAS Institute Inc., Cary, NC, US).

## Results

***Cohort characteristics***

In this cohort of children/youth with an ASD diagnosis (n_unweighted_ = 660, n_weighted_ = 112966), 80.5% were male, 91.4% were born in Canada and 76.1% were White (Table 2). The median household size where the child/youth resided was 3.6 people, and the median household income was $79770.

**Table 2:** Cohort characteristics of children/youth aged 5 to 17 years with an ASD diagnosis,a Canada, 2019

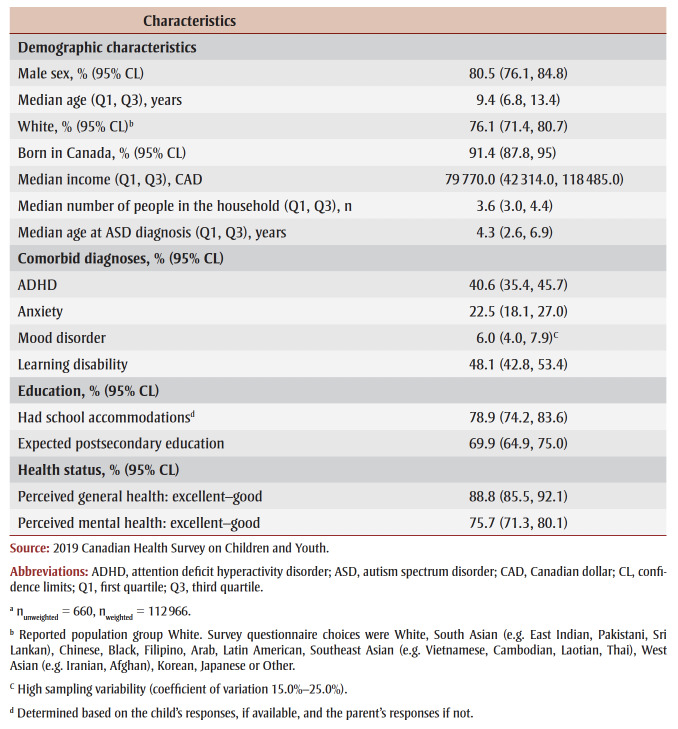

The median age at the time of the survey was 9.4 years and at time of ASD diagnosis was 4.3 years. More than one-third (40.6%) of the children/youth also had an ADHD diagnosis, while 22.5% were diagnosed with anxiety disorders and 6% with mood disorders (note: high sampling variability, i.e. CV between 15.0% and 25.0%). Almost half (48.1%) had a learning disability. 

Of those children/youth who attended school, 78.9% had academic accommodations and 6.8% of respondents were unsure if the child had accommodations (data not shown). In the case of 69.9% of the children/youth, their parents expected future postsecondary education. 

***Proportion of different functional difficulties***

Of all children/youth aged 5 to 17 years with an ASD diagnosis, 22.2% (95% CI: 17.9–26.5) reported functional difficulty with remembering/concentrating, 18.9% (95% CI: 14.7–23.0) with communicating and 13.3% (95% CI: 9.7–16.9) with self-care (Table 3). Functional difficulties with walking, seeing and hearing were less common.

**Table 3:** Percentage of functional difficulties in children/youth aged 5–17 years with an ASD diagnosis, Canada, 2019

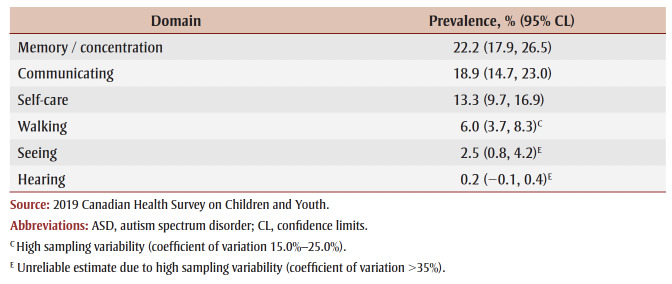

***Functional difficulty with remembering/concentrating***

Children/youth with ASD and functional difficulties with remembering/concentrating (n_unweighted_=650, n_weighted_=112037) were more likely to have a comorbid ADHD diagnosis (59.8% versus 35.4%, *p*< 0.001) and a learning disability (70.0% versus 42.1%, *p* < 0.001), and less likely to have a parental expectation of postsecondary education (54.3% versus 74.5%, *p*< 0.001) and good-to-excellent perceived general health (78% versus 91.9%, *p*<0.001) and mental health (56.4% versus 80.9%, *p* < 0.001), compared to those without this functional difficulty (Table 4).

**Table 4:** Significance testing for associated factors in children/youth aged 5–17 years with an ASD diagnosis, with and without remembering/concentrating functional difficulty, Canada, 2019

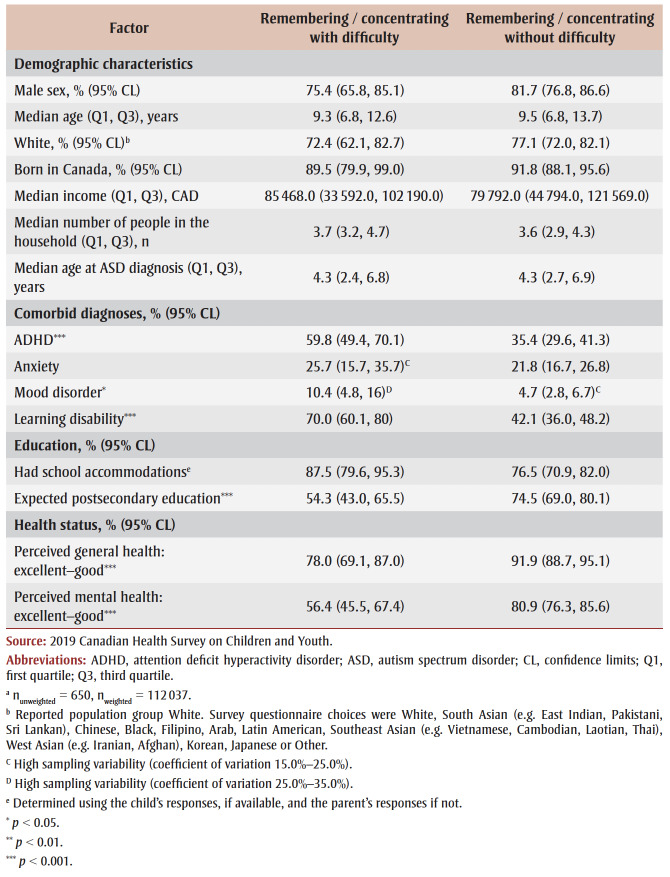

Having a comorbid ADHD diagnosis (odds ratio [OR] = 3.0; 95% CI: 1.5–5.9), learning disability (OR = 3.2; 95% CI: 1.5–6.7) and fair-to-poor perceived mental health (OR = 2.5; 95% CI: 1.2–5.2) were associated with higher odds of functional difficulty with remembering/concentrating among children/youth with ASD (Table 5).

**Table 5:** Results for multivariable logistic regression models of functional difficulties with communicating, remembering/concentrating and self-care among children/youth aged 5–17 years with an ASD diagnosis, Canada, 2019

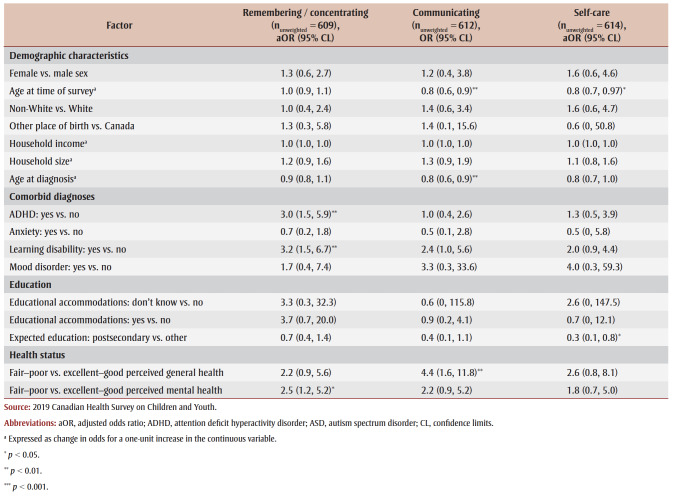

***Functional difficulty with communicating***

Children/youth with ASD and functional difficulties with communicating (n_unweighted_= 654, n_weighted_ = 112,366) were more likely to have a learning disability (63.2% versus 44.8%; *p* < 0.01) and less likely to be White (63.8% versus 78.7%; *p*< 0.01), have a parental expectation of postsecondary education (51.1% versus 73.8%; *p*<0.001) and report good-to-excellent perceived general health (74.3% versus 92.1%; *p*<0.001) and mental health (58.1% versus 79.9%; *p* < 0.001), compared to those without this functional difficulty with communicating (Table 6). 

**Table 6:** Significance testing for associated factors among children/youth aged 5–17 years with an ASD diagnosis, with and without communicating functional difficulty, Canada, 2019

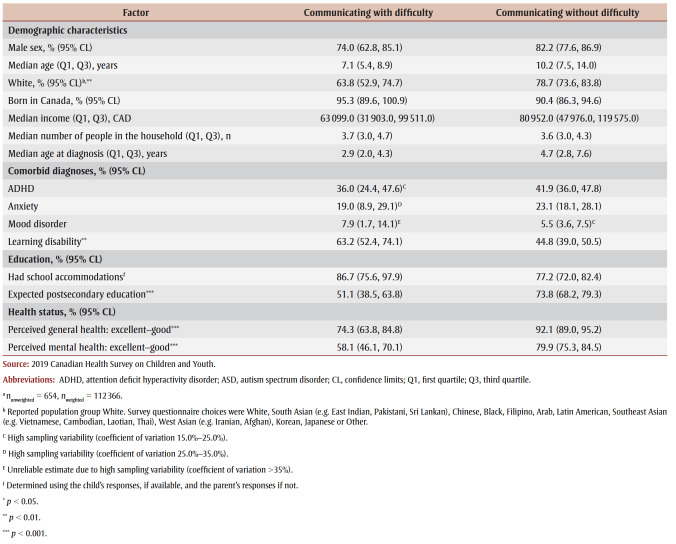

Older age at time of survey (OR = 0.8; 95% CI: 0.6–0.9) and at diagnosis (OR = 0.8; 95% CI: 0.6–0.9) were associated with lower odds of functional difficulty with communicating, and fair-to-poor perceived general health (OR = 4.4; 95% CI: 1.6–11.8) was associated with higher odds of functional difficulty ( Table 5).

***Functional difficulty with self-care***

Children/youth with ASD and functional difficulties with self-care (n_unweighted_ = 656; n_weighted_ = 112752) were more likely, compared to those who did not have difficulties with self-care, to have a learning disability (62.0% versus 46.0%; *p* < 0.05) and less likely to be male (69.6% versus 82.1%; *p*<0.05), have parental expectations of postsecondary education (44.5% versus 73.5%; *p*<0.001) and report good-to-excellent perceived general health (77.5% versus 90.6%; *p*<0.01) and mental health (59.7% versus 78.0%; *p* < 0.01) (Table 7). 

**Table 7:** Significance testing for associated factors in children/youth aged 5–17 years with an ASD diagnosis, with and without self-care functional difficulty, Canada, 2019

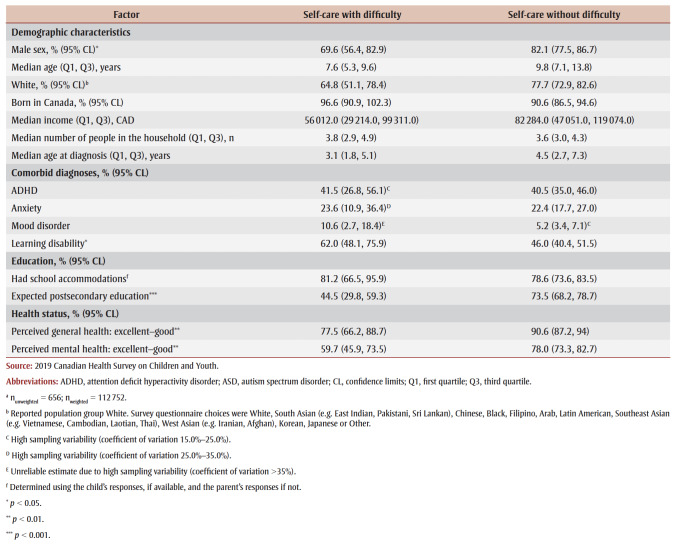

Increased age (OR=0.8; 95% CI: 0.7–0.97) and higher educational expectations (OR=0.3; 95% CI: 0.1–0.8) were associated with lower odds of functional difficulty with self-care (Table 5).

## Discussion

***Overall findings***

Our study investigated the prevalence, at the national level, of functional difficulties in Canadian children/youth aged 5 to 17 years diagnosed with ASD, focussing on difficulties with remembering/concentrating, communicating and self-care. We found that functional difficulties with remembering/concentrating (22.2%), communicating (18.9%) and self-care (13.3%) were the most common in this population. These rates demonstrate that children/youth with ASD share a diagnosis, but not necessarily the same functional abilities, suggesting that different functional ability profiles may be important for service delivery, clinical care and reporting.

Three out of five children/youth with ASD were found to have none of the functional difficulties included in the WG-SS. Even the most prevalent functional difficulty, remembering/concentrating, was only present in less than one in four of the children/youth with ASD. This indicates that having an ASD diagnosis does not directly translate to functional difficulties completing daily tasks, which supports previous research suggesting that adaptive functioning as well as symptom severity must be considered when studying developmental trajectories for children/youth with ASD.^31^

***Remembering/concentrating***

The finding that difficulties with memory and concentration are prevalent among children/youth with ASD is noteworthy, as these challenges are not typically considered core features of ASD. Previous research has reported higher prevalence of executive functioning difficulties and unique patterns of memory functioning among individuals with ASD.^32^-^34^ These findings suggest that executive function may be an important intervention target for children/youth with ASD. However, we also found that ADHD and learning disability diagnoses were associated with functional difficulties with remembering/concentrating. Both comorbidities are prevalent among children with ASD.^35^,^36^ The association of these comorbid characteristics with remembering/concentrating functional difficulty is difficult to disentangle because of our cross-sectional study design (see the “Limitations” section).

We found that perceived mental health was associated with functional difficulties with remembering/concentrating, after controlling for comorbid diagnoses and other individual characteristics. An individual’s functional capacity may influence the relationship between ASD symptoms and mental health; specifically, deficits in executive functioning, including working memory and cognitive flexibility, may exacerbate the mental health challenges of individuals with ASD. Prior research has proposed executive functioning skills as a potential pathway through which ASD symptoms in middle childhood are linked to mental health outcomes.^37^,^38^ These cross-sectional findings would be important for developing intervention programs to address challenges with memory and concentration. Incorporating strategies to improve executive functioning and memory skills within comprehensive intervention plans may contribute to better cognitive, adaptive, and mental health outcomes for children/youth with ASD.^39^,^40^

***Communicating***

The high prevalence of communication difficulties (1 in 5) observed in our study aligns with expectations, given that social and communication deficits characterize ASD.^16^,^41^ Communication difficulties often present significant barriers to social interaction and academic success for individuals with ASD,^42^,^43^ and interventions targeting communication skills are frequently prioritized as the first educational goal for ASD programs.^44^,^45^

We found an association between ASD diagnosis at an older age and decreased odds of functional communication difficulties. This may seem counterintuitive, given that early intervention has been shown to improve communication skills in children/youth with ASD,^46^‑^48^ and early diagnosis makes early intervention possible. However, it is imperative to distinguish between the causal relationships: more severe symptoms or functional difficulties may precipitate an earlier diagnosis. Earlier diagnosis of ASD has previously been associated with delays in social communication or the presence of an intellectual disability.^31^

Perceived general health was found to be strongly associated with functional difficulties with communication. Communication skills play a role in an individual’s ability to express their health care needs; in those with ASD, communication skills are a significant factor in successful health care interactions.^49^ However, it is essential to note that when perceived health is based on a parent’s perception, it may be influenced by the child’s inability to communicate effectively. Given the cross-sectional design, there is potential for bidirectional influences and confounding causes between perceived health and communication ability, particularly in this study. Further research using longitudinal data or experimental designs may help clarify these relationships and inform intervention strategies to improve communication and health care outcomes for individuals with ASD.

***Self-care***

When analyzing self-care functional difficulties, it is important to consider sensory issues, which are a common aspect of how individuals with ASD process and respond to sensory stimuli in their environments.^50^ These sensory challenges can contribute to difficulties with self-care activities, such as feeding and dressing,^51^ and addressing sensory needs in intervention programs designed for individuals with ASD is crucial to their overall development and well-being.^52^ By targeting and ameliorating sensory challenges, sensory-based interventions can enhance individuals’ ability to engage in self-care activities, thereby promoting greater independence and improved quality of life in children/youth with ASD.^53^

We also found that functional difficulties with self-care were associated with lower parental expectations for educational attainment. Although self-care activities such as feeding and dressing may not directly influence academic achievement, providing support beyond academic accommodations may increase the likelihood of success for planning to attend postsecondary education.^54^ Intervention programs can better equip children/youth with ASD for a successful transition to higher education by addressing sensory challenges and self-care difficulties and promoting more favourable long-term outcomes.

***Strengths and limitations***

Our study had several strengths. First, it was based on a dataset that, using sampling weights, closely represented Canadian children/youth aged 5 to 17 years living in private dwellings. Data from the CHSCY are nationally representative, providing greater coverage than previous geographically limited clinical studies. Second, there were few missing answers for individual questions, and sample weights were used to compensate for differences in response rates.^11^

Third, using the WG-SS allows for transferability of our results and for comparisons to other countries, disabilities and age groups. Fourth, by avoiding technical terms these functional difficulty measures were designed to be self-reported, making the WG-SS framework well-suited to survey data. In addition, the WG-SS questions are not specific to ASD, which means no assumptions regarding which difficulties might be the most common were made before data analysis.

Our study also had several limitations. First, the CHSCY is cross-sectional, which does not permit causal inferences. This limitation is important, especially when interpreting the relationship between functional difficulties with communicating and early diagnosis and discerning potential bidirectional associations. Longitudinal data collection could allow for future studies examining causality and influence.

Second, children/youth living on First Nations reserves and other Indigenous settlements in the provinces and in foster homes as well as institutions are excluded from the CHSCY sample, limiting the generalizability of the findings to all Canadian children/youth with ASD. Third, we observed high variability (CV >15%) in some estimates due to a relatively small number of sampled individuals. This high variability reduces the utility of some descriptive statistics.

Fourth, the study relied on the parent’s perception of a child’s difficulty, which cultural factors may influence.^55^ This reliance on parent/self-report could lead to results distorted by respondent bias or incorrect knowledge. Fifth, there is no formal validation of the ASD diagnosis that forms the basis for the studied subgroup. Sixth, the study can only claim to look at children/youth with an ASD diagnoses, not all children/youth with ASD, and the ability and inclination to access diagnosis are not evenly distributed.^56^

Seventh, while the WG-SS is broad, it does not include representations of all types of difficulty associated with disability. For example, symptoms of mental illnesses are not well-captured.^57^ Some of the WG-SS short-set domains are related to core indicators of ASD (e.g. communicating), but others are not. Common functional difficulties for children/youth with ASD, including social interaction and controlling behaviour, are not part of the WG-SS. Thus, this study does not comprehensively cover all possible difficulties with functioning—only the six in the WG-SS, with a focus on remembering/concentrating, communicating and self-care. Further, results should be interpreted with caution due to the lack of WG-SS validation specifically for children/youth with ASD. Future development of a survey-compatible measurement tool specific to children/youth with ASD would allow for more sophisticated analysis of these children/youth’s difficulties. 

Eighth, our cohort does not include children younger than 5 years who may have a higher prevalence of these functional difficulties given more severe symptoms are often associated with an earlier ASD diagnosis. 

Finally, the COVID-19 pandemic may have affected the day-to-day functioning and health of children/youth with ASD. As such, the estimates provided here may not reflect the current rates of functional difficulties. We aim to update these estimates once the next cycle of CHSCY is released, in summer 2024.^58^

## Conclusion

Our study highlights the variable prevalence of certain functional difficulties in Canadian children/youth aged 5 to 17 diagnosed with ASD and identifies important factors associated with these functional difficulties in this population. Together, these findings suggest that an ASD diagnosis does not necessarily mean a child/youth will experience functional difficulties and emphasize the need for targeted and personalized intervention programs to address challenges. 

Our findings are only a first step towards understanding the specific challenges that children/youth with ASD face: more specialized measurement tools and longitudinal data collection are required to understand the full range of functional abilities and the underlying mechanisms involved.

## Acknowledgements

We would like to thank those at Statistics Canada who designed the 2019 Canadian Health Survey on Children and Youth (CHSCY) and those who collected and processed the data. We would also like to express our gratitude to the respondents of the CHSCY, without whom this work would not have been possible.

## Funding

None.

## Conflicts of interest

None.

## Authors’ contributions and statement

AF – Methodology, formal analysis, interpretation of the results, writing – original draft, writing – review and editing

AA – Methodology, formal analysis, interpretation of the results, writing – original draft, writing – review and editing

SO – Conceptualization, methodology, writing– review and editing

SP – Conceptualization, methodology, writing – review and editing

SG – Writing – review and editing

JYC – Writing – review and editing

PM – Writing – review and editing

RE – Conceptualization, project administration, methodology, supervision, writing – review and editing

The content and views expressed in this article are those of the authors and do not necessarily reflect those of the Government of Canada. 
